# Using *in vivo* functional and structural connectivity to predict chronic stroke aphasia deficits

**DOI:** 10.1093/brain/awac388

**Published:** 2022-11-08

**Authors:** Ying Zhao, Christopher R Cox, Matthew A Lambon Ralph, Ajay D Halai

**Affiliations:** MRC Cognition and Brain Sciences Unit, University of Cambridge, Cambridge, UK; Department of Psychiatry, University of Cambridge, Cambridge, UK; Department of Psychology, Louisiana State University, Baton Rouge, LA, USA; MRC Cognition and Brain Sciences Unit, University of Cambridge, Cambridge, UK; MRC Cognition and Brain Sciences Unit, University of Cambridge, Cambridge, UK

**Keywords:** stroke aphasia, functional connectivity, resting-state fMRI, structural connectivity, diffusion tensor imaging

## Abstract

Focal brain damage caused by stroke can result in aphasia and advances in cognitive neuroscience suggest that impairment may be associated with network-level disorder rather than just circumscribed cortical damage. Several studies have shown meaningful relationships between brain–behaviour using lesions; however, only a handful of studies have incorporated *in vivo* structural and functional connectivity. Patients with chronic post-stroke aphasia were assessed with structural (*n* = 68) and functional (*n* = 39) MRI to assess whether predicting performance can be improved with multiple modalities and if additional variance can be explained compared to lesion models alone. These neural measurements were used to construct models to predict four key language-cognitive factors: (i) phonology; (ii) semantics; (iii) executive function; and (iv) fluency. Our results showed that each factor (except executive ability) could be significantly related to each neural measurement alone; however, structural and functional connectivity models did not explain additional variance above the lesion models. We did find evidence that the structural and functional predictors may be linked to the core lesion sites. First, the predictive functional connectivity features were found to be located within functional resting-state networks identified in healthy controls, suggesting that the result might reflect functionally specific reorganization (damage to a node within a network can result in disruption to the entire network). Second, predictive structural connectivity features were located within core lesion sites, suggesting that multimodal information may be redundant in prediction modelling. In addition, we observed that the optimum sparsity within the regularized regression models differed for each behavioural component and across different imaging features, suggesting that future studies should consider optimizing hyperparameters related to sparsity per target. Together, the results indicate that the observed network-level disruption was predicted by the lesion alone and does not significantly improve model performance in predicting the profile of language impairment.

## Teaser

Zhao *et al.* use *in vivo* structural and functional connectivity to predict phonology, semantics, executive function and fluency in patients with aphasia. While both modalities can predict these behavioural components, they do not explain deficits over and above a model using T1 lesion information.

## Introduction

Speech and language problems (aphasia) are a disabling feature of chronic post-stroke survivors,^1^ present in around one-third of cases.^2^ A recent study highlighting the global burden of stroke found that mortality and incidence rates are declining, although the former has done so at a higher rate.^3^ This results in large numbers of survivors needing long-term care and rehabilitation, thus creating a need to improve clinical diagnosis, prognosis, management and therapy pathways. Several research groups have used neuroimaging methodologies to predict aphasia related behavioural performance and/or recovery following stroke, where most studies use lesions from structural MRI scans.^4–8^ It is becoming clear that, while prediction models using lesion data alone are producing promising results, there is still substantial remaining variance to be explained, making clinical translation difficult. This has spurred recent studies to focus on neural measures other than lesion locations such as structural connectivity (SC),^9–13^ functional properties of the remaining tissue^14–16^ or both in parallel.^17,18^ Critically, given that data collection in this population is challenging, not all studies have used *in vivo* measures of SC and/or functional connectivity (FC) when building models to predict the severity of impairments/recovery in post-stroke aphasia. Therefore, the primary aim of this study was, for the first time, to create prediction models across three *in vivo* modalities; T_1_ (lesion site), diffusion weighted SC and resting-state FC.

Classical neurology strived to understand brain-behavioural relationships in terms of specific functional nodes critical for certain behaviours.^19,20^ In parallel, it was posited that behaviours are supported by brain networks where nodes (regions) communicate (FC) via white matter fibres (SC). This theory dates back to classical neurology^20–22^ and has been reinvigorated over the last few decades due to advances in neuroimaging techniques.^23–29^ Despite the importance of SC and FC, there are only a handful of studies that have explored their relationship to behavioural performance and recovery using *in vivo* measures in stroke aphasia.^9–11,14,17,30^ This is partly related to the challenges of collecting large scale multimodal imaging data in stroke populations, which has led to the development of pseudo-measures based on ‘damaging’ healthy datasets.^12,18^ While several studies have reported significant improvements using multimodal measures,^9,10,18^ others have failed to replicate.^12,13^ For example, a recent study by Halai *et al*.^13^ did not find model improvements by incorporating fractional anisotropy, mean diffusivity and anatomical connectivity data. Similarly, a study by Del Gazio *et al*.^17^ reported that white matter connections did not improve models beyond the lesion but, by adding graph theory measures, the models did improve significantly albeit by a small amount (note model correlations were *r* = 0.72 versus 0.76, respectively). One explanation for the lack of model improvement comes from the finding that the lesion and connectivity profiles are highly correlated (*r* = 0.94 between first principal components).^12^ Accordingly, the current study: (i) used *in vivo* FC and SC measures in a chronic post-stroke aphasic sample; and (ii) compared models using connectivity features with lesion models across the whole brain to determine whether either connectivity modality can explain variance over and above the lesion models.

Finally, one caveat in using neuroimaging data in these types of analyses is related to the ‘curse of dimensionality’. Although progress has been made to obtain larger samples,^4^ the number of predictors/features far outweigh sample size. There is a methodological challenge, therefore, to use appropriate techniques that allow for suitable mapping but also retain cognitive interpretation. For example, FC was mapped to behavioural deficits in a stroke population using a ridge regression model but the overall results were discussed on the inter- and intra-hemispheric scale.^14^ Ridge regression is one of three regularized least-squares methods, which includes elastic net and least absolute shrinkage and selection operator (LASSO).^31^ Ridge regression models are dense (every feature is given a weight), while LASSO models are sparse (only a small number of connections are given a weight) and elastic nets are in between the two. Each method applies penalties in an attempt to control for overfitting but, where there are a large number of features, the LASSO models are potentially more cognitively interpretable since these models actively reduce the number of features. To date, studies tend to use a specific form (or set level) of penalty and apply this to every target/behaviour. For example, Siegel *et al*.^14^ applied ridge regression to all models, in contrast to Pustina *et al*.,^32^ where sparsity was optimized for each model. In this study, we observed the overall level of sparsity for each behavioural target. We focused on predicting behavioural component scores, which have been shown to describe a range of stroke deficits in acute^33^ and chronic patients.^8,14,34–39^ In addition to using sparse models to help improve interpretation of the models, we also used a graph theory metric, nodal degree, to summarize connectivity strength at each node.^40^ This method can simplify a model and provide an intuitive way of summarizing the connection weights to aid interpretation.^14^

## Materials and methods

### Subjects

Seventy participants with chronic post-stroke aphasia took part in the study. Many have been included in previously published studies.^7,8,34,36,37,41,42^ The criteria for recruiting patients into the database included: (i) monolingual native English speakers; (ii) normal or corrected-to-normal hearing and vision; (iii) right-handed; (iv) single known stroke; (v) at least 12 months post-stroke; (vi) no other known neurological conditions; and (vii) no contraindications for MRI scanning. We included subjects spanning the range of aphasia severity (from global to well-recovered). Informed consent was obtained from all participants under approval from the local ethics committee (North West Haydock MREC 01/8/94). All subjects underwent detailed behavioural assessment and a subset completed the neuroimaging protocols: high resolution T_1_-weighted scan (all), diffusion weighted imaging (DWI) (*n* = 68), and resting-state functional MRI (rs-fMRI; *n* = 39). To use the maximum amount of data possible for each analysis, subsequent models were created using different sample sizes [i.e. voxel-based correlational methodology (VBCM) *n* = 68, DWI *n* = 68 and rs-fMRI *n* = 39; combined DWI-rsfMRI *n* = 39]. Structural imaging data from a healthy age and education matched control group (10 female, 12 male) were used to determine abnormal tissue in the patients using an automated lesion identification toolbox.^43^ We used rs-fMRI data from a younger control sample (*n* = 30, age range = 25–43, mean = 30.5 and SD = 5.0) acquired with the same dual echo fMRI protocol as the stroke patients. While it may have been possible to use publicly available data, we specifically used an fMRI acquisition protocol designed to improve signal-to-noise in the anterior temporal and orbitofrontal regions (see the ‘Acquisition of neuroimaging data’ section for more details).

### Neuropsychological assessments and analysis

To test the participants’ speech, language and cognitive abilities, we obtained scores on a large neuropsychological test battery (see Supplementary material, section 1 for full details). The battery was designed to assess input/output phonological processing, semantic processing and sentence comprehension, as well as general cognitive function and has been used in previous publications by our research group..^34,36^ All scores were converted into percentage based on the maximum score available; where no maximum was available, we used the max score in the group. The scores were then used to identify latent cognitive factors, as shown previously.^34,36^ We performed a varimax rotated principal component analysis, where factors with eigenvalues <1.0 were excluded (based on Kaiser’s rule stating that the retained components have greater or equal power to explain the data than a single variable^44^). The matrix coefficients were used to project factor scores for each individual, which were in turn used as targets during subsequent analyses.

### Acquisition of neuroimaging data and preprocessing

Given the multimodal nature of data collected for this study, we provide detailed information in the Supplementary material, section 2 for each protocol and the specific preprocessing steps. In summary, we collected high resolution structural T_1_-weighted imaging, resting-state fMRI and DWI using a 3T Philips Achieva scanner (Philips Healthcare).

T_1_-weighted images were preprocessed with the same procedure as our previous studies^34,36^ using Statistical Parametric Mapping software (SPM8: Wellcome Trust Centre for Neuroimaging, http://www.fil.ion.ucl.ac.uk/spm/) and a modified segmentation-normalization procedure.^43^ In brief, this method allows us to identify abnormal voxels (based on a control population), which was used as a cost function mask during Montreal Neurological Institute (MNI) normalization. Images in standard space were smoothed with an 8 mm full-width at half-maximum Gaussian kernel. All resultant lesion maps were visually inspected and any discrepancies were manually corrected.

We used a specialized dual gradient echo planar imaging technique for the rs-fMRI protocol to improve signal detection within inferior temporal and orbitofrontal regions.^45–47^ We collected data from the whole brain and participants were instructed to lie still and look at a fixation cross during scanning. The rs-fMRI data were processed using SPM8 and the Data Processing Assistant for Resting-State fMRI (DPARSF Advanced Edition, v.2.3) toolbox.^48^ The raw data were subjected to the following processes (see Supplementary material, section 2 for specific details): basic image preprocessing, estimation of nuisance variables and removal,^49,50^ MNI normalization using native-to-MNI transforms from the Seghier *et al*.^43^ method, 8-mm full-width at half-maximum smoothing, removal of linear trend and bandpass filtering (0.01–0.08 Hz).

The diffusion data were acquired in two runs using reverse phase encoding (L–R and R–L), where each run consisted of 43 non-collinear diffusion directions at *b* = 1200 s/mm^2^, covering the whole brain.^51^ Processing of the diffusion tensor imaging data was conducted using FSL’s (v.5.0.10) diffusion pipeline^52^ (see Supplementary material, section 2 for full details). The data were prepared and submitted to FSL’s TOPUP and EDDY tool to correct susceptibility inducted distortions^53^ and distortions related to eddy currents and head motion.^54^ The Bayesian Estimation of Diffusion Parameters Obtained using Sampling Techniques (BEDPOSTX) method was used to estimate fibre orientation estimate fibre orientation distributions at each voxel.^55,56^

### Using T_1_ to predict the behavioural factors

**Figure 1:**
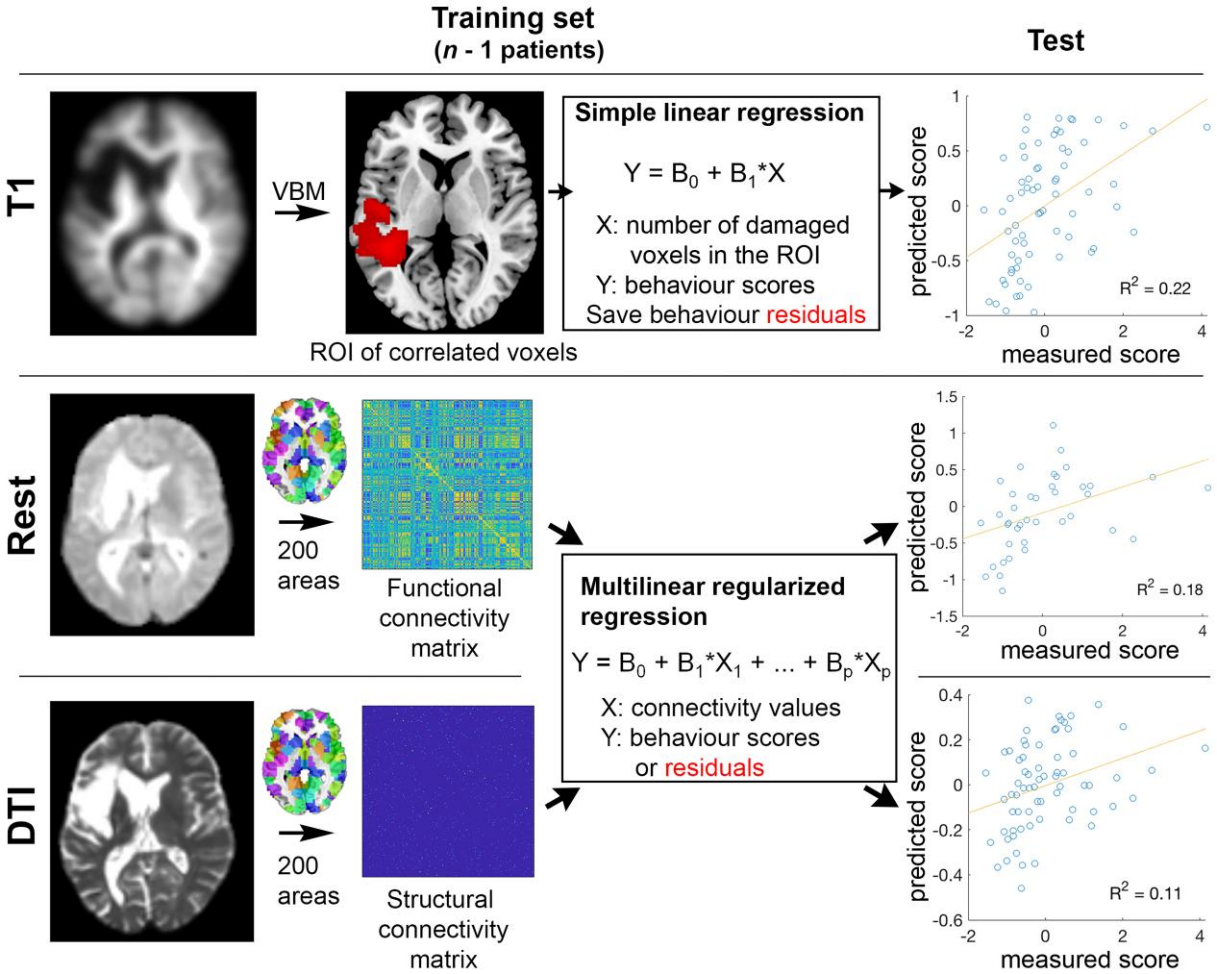
**Illustration of the analyses pipeline.** T_1_, FC from rs-fMRI, SC from DWI were used to predict behavioural factor scores in a LOOCV. FC and SC were also used to predict the residual behavioural scores.

The normalized and smoothed T_1_ images were used to predict the behavioural factors scores with 68 patients (who had both T_1_ and diffusion MRI data). Patients were separated into training and test sets on the basis of leave-one-out cross-validation (LOOCV) (see Fig. 1 for illustration). We performed a whole-brain VBCM^57^ analysis using the training set factor scores, which were correlated to the T_1_ data. Significant clusters were used to define regions of interest (ROI) for each factor score using the following threshold: voxel-wise false-discovery rate corrected *P* < 0.05 and cluster size >2 cm^3^, as we wanted to correct for multiple comparisons but avoid small clusters. All surviving ROI were used to determine lesion load. A linear regression model was then built within the training set relating the patients’ lesion load in the ROI and each behavioural factor. Finally, we calculated the lesion load for the left-out case and projected that data through the regression model built on the training set to obtain a predicted score for the left-out case (maintaining full separation between training and test data). This was repeated for each fold to obtain a predicted score per subject. Model accuracy was indicated by Pearson correlation between lesion predicted scores and the cross-validated behavioural factor scores. A null distribution was obtained using a permutation test (shuffling the behavioural factor scores and refitting the model each time) to test for significance (*n* = 1000). The residual scores of each training set and test set were saved for the later analysis.

### Functional and structural connectivity estimation

We used the Craddock atlas consisting of 200 brain regions^58^ to compute an average timeseries within each ROI. This atlas was developed using resting-state data but the same atlas was used for FC and SC to directly compare the models. We note that an alternative atlas,^59^ which is based on different parcellation procedure, produced similar results but are not discussed further.

For the functional data, we first extracted the mean time course within each ROI and then calculated pair-wise Pearson’s correlations to every other ROI (resulting in 19 900 connections for each patient as the matrix is symmetrical). The correlation matrix was Fisher *z-*transformed. To minimize the effects of lesions on the multivariate models, we set the correlation/connection value between any ROI pair that had overlap with the subject’s lesion (regardless of amount, i.e. >1 voxel) to zero (i.e. no valid correlation/connection can be obtained emanating from the lesioned area).^14^

For the structural data, we first transformed the Craddock atlas into native diffusion space, using the inverse MNI transform (to native T_1_ space) followed by a linear transform to diffusion space using FMRIB’s Linear Image Registration Tool.^60^ All tracking was performed using the ‘Pipeline for Analyzing braiN Diffusion imAges’ (PANDA)^61^ software, which automates diffusion analyses as well as processes subjects in parallel on a distributed computer. We used probtrackx2 command to seed from each ROI. For each voxel in the seed ROI, 5000 streamlines were initiated with the following parameters: curvature threshold 0.2, 1000 maximum steps and step length 0.5 mm, loop check and correcting path distribution for the length of pathways. We calculated the probabilistic connectivity from seed to target as the number of streamlines arriving at the target divided by the total streamlines sent by the seed ROI. To minimize the effect of lesion on the multivariate models, connections emanating from an ROI within a lesion (>95% damage) was set to zero although in general tracking through lesioned space in diffusion data self-terminates. The resulting matrix is not symmetric, therefore, we averaged the two reciprocal connections^61^ resulting in 19 900 connections per subject.

### Building multivariate models to predict behaviour factor scores

A single matrix was produced for each modality to facilitate modelling. Each matrix had a row for each patient and a column for each variable. Each variable represented either a voxel (in the lesion map) or a relationship between a pair of ROI (in the SC and FC matrices). As noted before, the FC and SC matrices were symmetric, therefore we extracted values below the diagonal before collapsing to a vector per subject.

We then used elastic net regularized regression to predict behavioural factor scores based on the lesion or connectivity profiles. Regularized regression constrains the model’s degrees of freedom by applying a penalty that is proportional to the magnitude of the regression coefficients. The ridge penalty is determined by the sum of squared coefficients, and the LASSO penalty is determined by the sum of their absolute values. The best ridge solutions diffuse weight over correlated variables, rather than putting a large coefficient on any single variable. Conversely, the best LASSO solutions are sparse. Correlated variables are assigned zero weight while large weights (relative to ridge regression) are allocated to the fewest number of variables that account for independent variance. Both approaches are therefore appropriate for problems with more variables than examples to model, which cannot be solved without some constraint on degrees of freedom. When applying either ridge or LASSO, the severity of the constraint on degrees of freedom is controlled by a hyperparameter *λ* that is tuned to the data. However, as the structure of the brain is neither excessively sparse nor densely connected, neither the ridge nor LASSO penalty will discover models that have a desired level as structured sparsity. The elastic net penalty dynamically combines the ridge and LASSO penalties to best fit the data. In addition to the hyperparameter *λ*, a hyperparameter *α* controls what proportion of the constraint should come from the ridge penalty or the LASSO penalty. The best elastic net solutions will identify a sparse collection of groups of correlated variables. Such solutions tend to correspond to more accurate and interpretable models of the data. We implemented our models using GLMNet^62^ in MATLAB (2014a; The MathWorks, Natick, MA, USA), which efficiently and automatically handles the search for *λ* and we tuned *α* via nested LOOCV.

We fitted models to each of the four behavioural components separately, based on the SC data, the FC data and both simultaneously (12 models in total). Model performance was assessed through LOOCV, where on each fold the connectivity data for the left-out case were used to predict the factor score. We computed Pearson’s correlation between these and the target (true) factor scores. The statistical significance of these correlations was determined by permutation test (*n* = 1000): repeat the LOOCV procedure by shuffling the target scores. Statistical inference was determined using a statistical criterion of *α*=0.05. The beta weights for each connection were averaged in the final model (for each behavioural component) across all cross-validated folds and projected into brain space for visualization (applying a *z*-transform). Each model had many connections making them difficult to interpret; therefore, we calculated the nodal degree (sum of all weights per node),^40^ to simplify the model. For ease of visualization, we show the top 10 nodes, where for each node we also determined the proportion of positive and negative connections. As a final step, we filtered the original connectivity map to show connections emanating only from the top 10 nodes (we note that this filtering strategy by definition is not the same as the original model, it is purely a way to simplify the model to aid interpretation). All brain maps were plotted using BrainNet Viewer.^63^

For completeness, we also repeated the multivariate analyses above using the voxel-wise T_1_ data (*n* = 68). For clarity, we report the VBCM results in the main paper and report the multivariate results in Supplementary material, section 5 (see the ‘Results' section for details).

### Comparing connectivity and lesion models

We explored whether the connectivity measures could explain variance over and above T_1_ (lesion) data in two ways. We first compared the connectivity models and lesion models (VBCM) directly by comparing the squared prediction error with a Wilcoxon sign rank test^14^ for the subset of data with complete imaging (i.e. *n* = 39). Second, we took the residual scores from the lesion models (i.e. variance unexplained) and used these scores as targets for the connectivity models (using the maximum *n* samples available in each modality). The predictive models were built in the same way as noted previously.

### Functional connectivity of lesion predictors

We used a rs-fMRI dataset composed of healthy control participants to generate maps of the typical FC for each behavioural factor using the lesion ROI as seeds (using only grey matter). The FC maps were Fisher *z*-transformed and all maps relating to each factor score were subjected to a one-sample *t*-test (voxel *P* < 0.001, cluster corrected *P* < 0.05 with AlphaSim) to generate a group network for each factor score. To compare the similarity between the patient results and control networks, we took the top 10 predictive nodes from the patient data and overlaid them with the FC maps generated with the control rs-fMRI data.

### Data availability

Behavioural data are available in Supplementary material, section 3. Further data are available by request to M.A.L.R.

## Results

### Neuropsychological and lesion distribution

**Figure 2:**
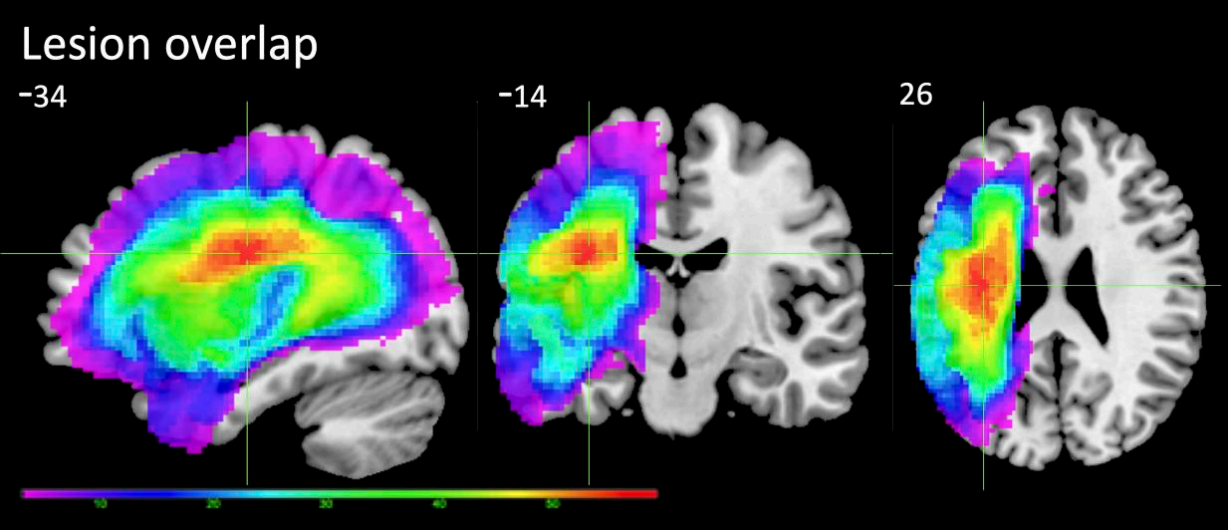
**Lesion overlap map for 68 patients in the study.** The figure represents the number of cases with damage to a voxel (scale 3–59). The voxel with maximum lesion is show with the crosshairs.

A summary of behavioural scores for the 70 cases is provided in Supplementary material, section 3. The sample contained the full range of severity from global aphasia to well-recovered cases. A lesion overlap map for the 68 stroke aphasic participants that were used in either the FC/SC regression analysis is provided in Fig. 2 and primarily covers the left hemisphere area supplied by the middle cerebral artery.^64^ The maximum number of participants who had a lesion in any one voxel was 59 (MNI coordinates −34, −14, 26, the anatomy of peak: the left superior longitudinal fasciculus).

### Behavioural components

The factor analysis on the behavioural data of 70 patients revealed four distinct dimensions (Supplementary material, section 4), as reported previously in this dataset.^36^ The degree to which each test loaded on the components allowed us to interpret the cognitive meaning of each component as follows: (i) phonological ability, due to high loadings from repetition, naming, and digit span tests; (ii) semantic processing, due to high loadings from picture matching, synonym judgement, Camel and Cactus, and type/token ratio tests; (iii) executive cognition, due to high loadings from minimal pairs, Raven’s coloured progressive matrices and the Brixton spatial anticipation tests; and (iv) fluency, due to high loadings from tests assessing the number of speech tokens, words per minute and mean length of utterances.

### Using T_1_ to predict the behavioural factors

**Figure 3:**
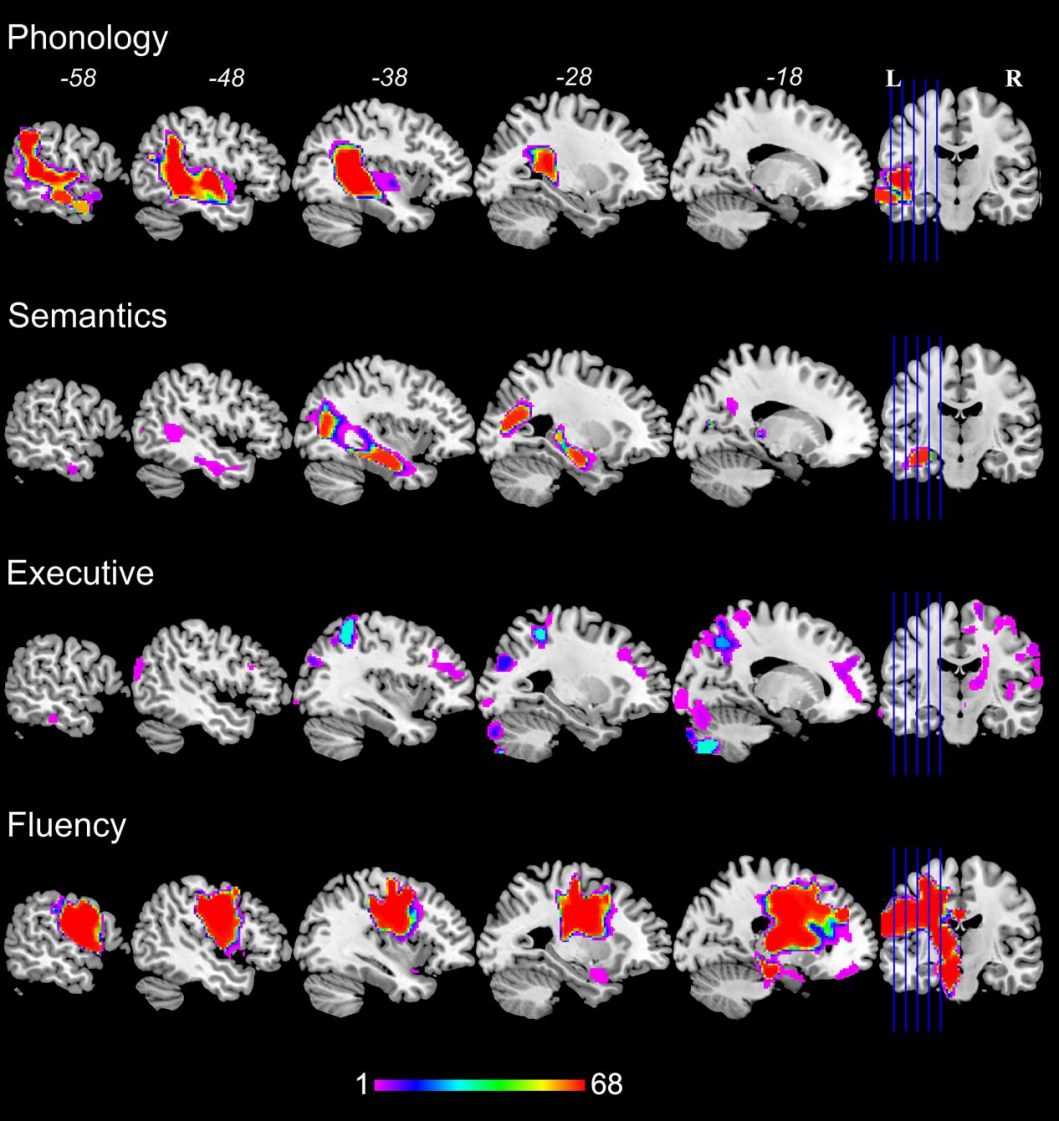
**Neural correlates of behavioural components.** Neural correlates of principal component scores related to phonology, semantics, executive function and speech fluency using LOOCV VBCM (68 patients). For each loop, the threshold was voxel false-discovery rate corrected *P* < 0.05 and cluster >2 cm^3^. The significant regions from each loop were overlapped and mapped on the brain. Hot colours indicate a consistent/stable mapping with the behaviour across all loops.

The regression models using the T_1_ lesion maps could predict three out of four factor scores: phonology (*r* = 0.41, *P* = 0.001), semantics (*r* = 0.38, *P* = 0.004), executive function (*r* = −0.08, ns) and fluency (*r* = 0.46, *P* = 0.001). In Fig. 3, we show the relative stability of the clusters that are selected across all LOOCV iterations. The most stable clusters for phonology were located at the superior temporal gyrus and extended to the supramarginal gyrus. The semantic factor was related to medial and anterior parts of the temporal lobe, as well as the underlying white matter (inferior longitudinal fasciculus). The fluency factor related with precentral and postcentral gyri, as well as underlying white matter (i.e. corticospinal tract and frontal aslant tract). We did not find stable clusters for the executive component, consistent with poor model performance. We note that we also applied a regularized regression approach to the T_1_ lesion data, with largely similar results to those reported before with the exception of the executive component (Supplementary material, section 5).

### Regularized regression models predicting behaviours

**Figure 4:**
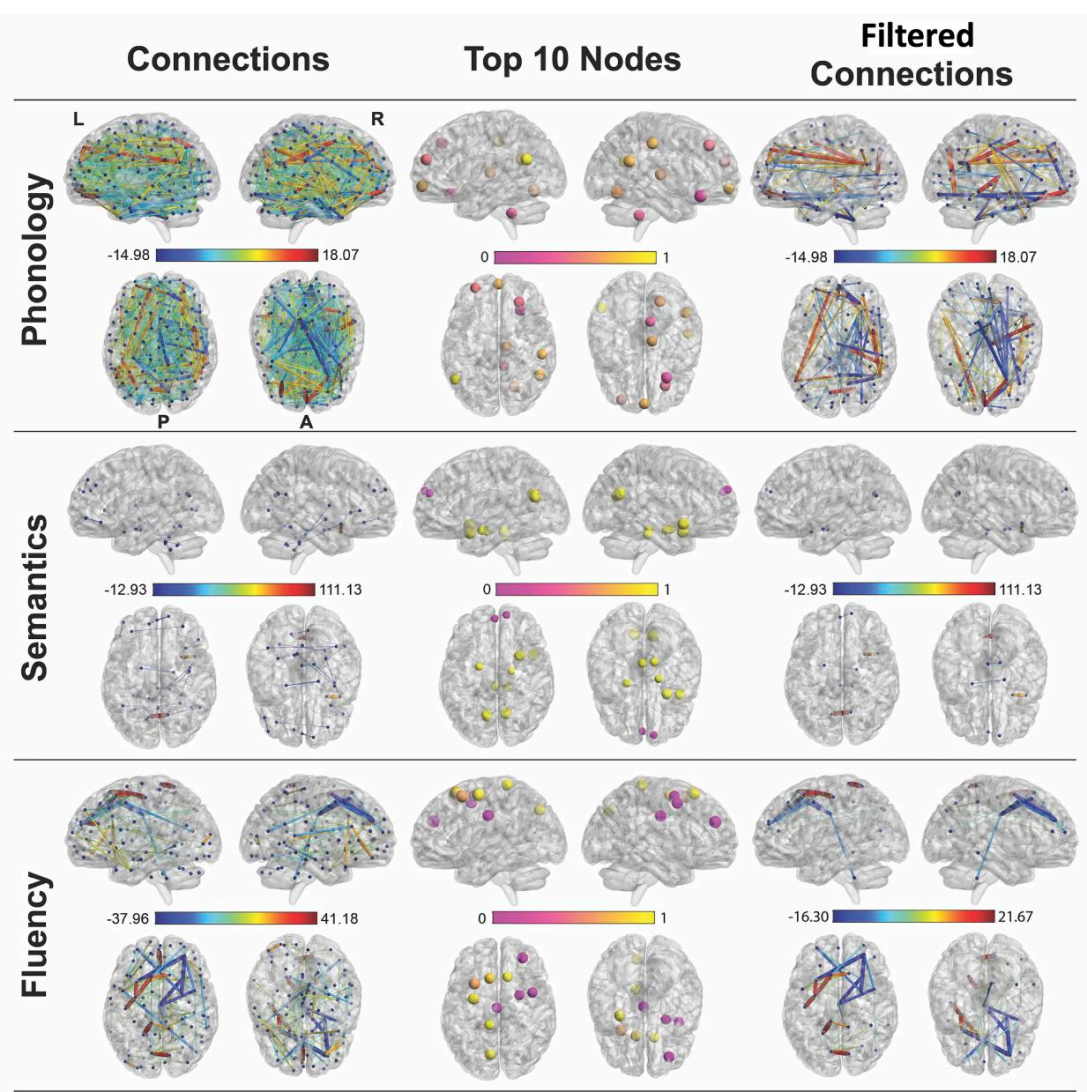
**FC models predicting behavioural components.** Regularized regression model of FC for phonology (*top row*), semantics (*middle row*) and fluency (*bottom row*). The *leftcolumn* shows connections in the model that had *z*-weights >3.29 (*P* < 0.001) (averaged across all folds). The *middle column* shows nodal degree (sum of absolute weight at each node) (top 10 shown). The scale (0–1) indicates the proportion of positive weights. The *right column* shows the significant connections filtered through the top 10 nodes. L = left; R = right; P = posterior; A = anterior. Left is *left*.

**Figure 5:**
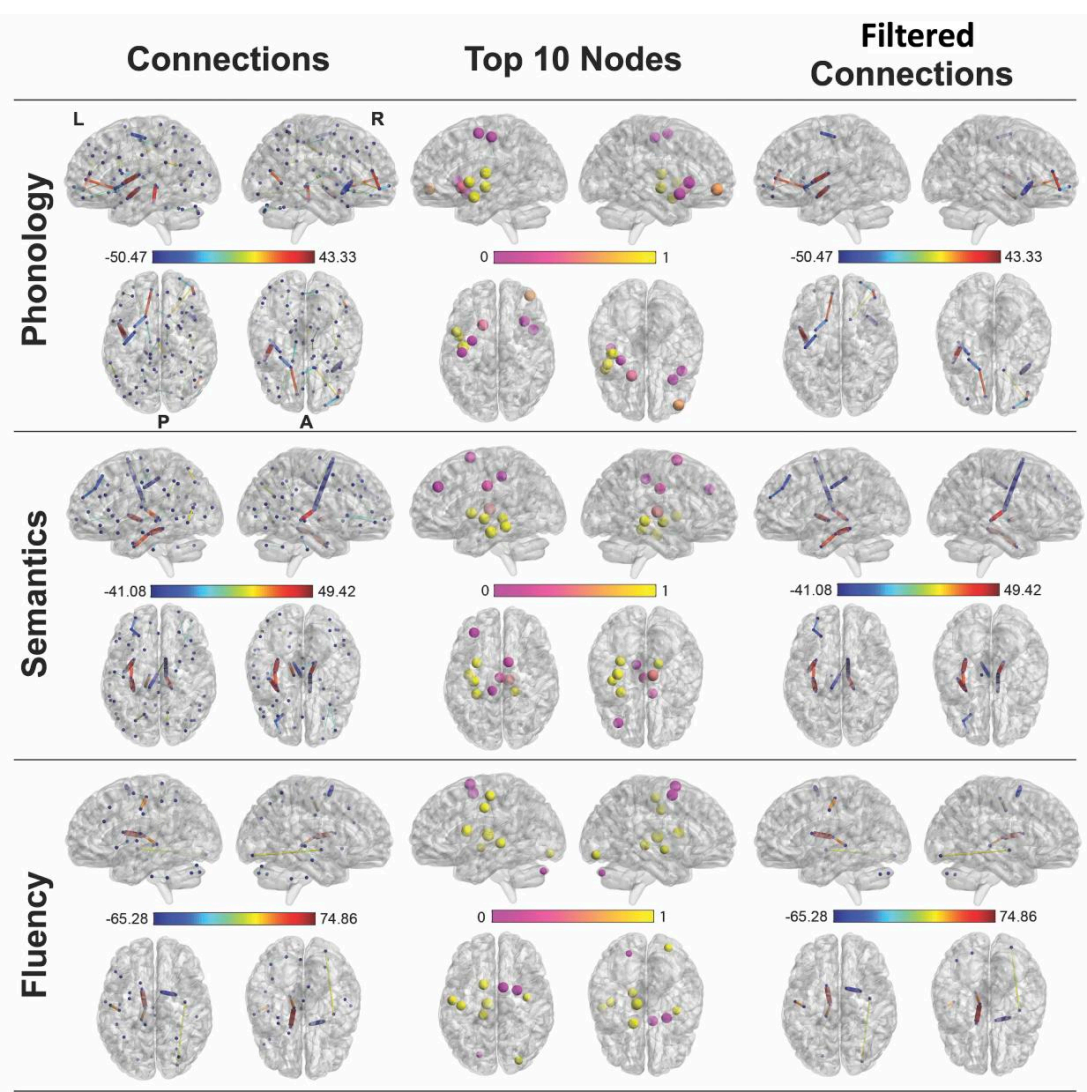
**SC models predicting behavioural components.** Regularized regression model of SC for phonology (*top row*), semantics (*middle row*) and fluency (*bottom row*). The ‘Connection’ brain figure (*left*) shows connections in the model that had *z*-weights >3.29 (*P* < 0.001) (averaged across all folds). The ‘Nodes’ brain figure (*middle*) shows nodal degree, which reflects the nodes with the highest cumulative absolute connection weights (top 10 shown). The scale 0 to 1 indicates the percentage of positive connections that were predictive. The ‘Filtered connection’ brain figure (*right*) shows the significant connections from the top 10 nodes as a way to filter the number of connections. L = left; R = right.

The following section provides model accuracy (Pearson’s correlation) for functional and structural connections, separately as well as jointly. A summary of model weights projected to brain space are shown in Figs 4 and 5.

#### Functional connectivity

FC data were available for 39/70 patients. We reproduced the pattern of results observed for the T_1_ data, where FC profiles predicted phonology (*r* = 0.35, *P* = 0.013, semantics (*r* = 0.50, *P* = 0.001) and fluency (*r* = 0.40, *P* = 0.005) but not executive function (*r* = −0.19, ns). A direct comparison between the squared prediction error between models using FC data and T_1_ showed that model performance was not significantly different for any target (Table 1); in addition, the FC predictors did not significantly predict the residual scores after accounting for lesion). Interestingly, the level of sparsity differed across behavioural components (distribution of alpha values is given in Supplementary material, section 6). Figure 4 displays three perspectives on the significant models. From left to right, the first column presents all edges included in the model (magnitude indicated by thickness). The second column presents the 10 nodes with the largest nodal degree, coloured to convey their relative proportion of positive/negative weights strength (0 = all weights were negative and 1 = all weights were positive). The third column presents a filtered view of the full model, specifically only connections emanating from the top 10 nodes.

**Table 1:** Comparing prediction accuracy (squared error) between connectivity models and T_1_-lesion model (on subset of sample *n* = 39)

	Median squared error (SD)				Model comparison					
					T_1_-SC		T_1_-FC		T_1_-FCSC	
Target	T_1_	SC	FC	FCSC	*z*-score	*P*	*z*-score	*P*	*z*-score	*P*
Phonology	0.27 (0.91)	0.62 (1.24)	0.39 (1.27)	0.44 (0.98)	**−3.50**	**4.6 × 10^−4^**	−0.57	0.567	−1.07	0.283
Semantics	0.24 (3.02)	0.34 (3.15)	0.24 (2.82)	0.26 (2.67)	−1.49	0.135	0.71	0.477	0.04	0.967
Executive	0.54 (1.52)	0.34 (1.89)	0.53 (1.41)	0.54 (1.44)	0.17	0.867	1.27	0.204	1.48	0.139
Fluency	0.22 (3.78)	0.32 (3.46)	0.22 (2.69)	0.29 (2.83)	−1.12	0.264	0.24	0.812	−0.63	0.530

For phonology, the model was ridge-like, with the strongest nodes residing in right frontal and parietal regions. All nodes were connected by a mixture of positive and negative weights. In contrast, a sparse model was observed for semantic ability, with prominent bilateral inter-hemispheric connections between the ventral temporal lobes and occipital lobe. This was reinforced with the filtered results, where positive nodes were located within bilateral ventral temporal and occipital regions. Finally, a moderately sparse model was observed for speech fluency, with most weight attributed to connections in bilateral frontal cortex, further confirmed by the location of the 10 strongest nodes. The nodes were connected by a mixture of positive and negative weights.

#### Structural connectivity

SC data were available for 68 out of 70 patients and the results are shown in Fig. 5. We found significant models for phonology (*r* = 0.29, *P* = 0.007), semantics (*r* = 0.20, *P* = 0.050), marginally significant for fluency (*r* = 0.17, *P* = 0.077) but not significant for executive function (*r* = 0.003, ns). Again, the level of sparsity varied across behavioural components (Supplementary material, section 6). A sparse model was observed for phonology (in contrast to a dense FC model), with prominent weights in the left superior temporal gyrus, inferior frontal gyrus and right frontal pole regions. For semantics, a moderately inclusive model was observed, with most weight attributed to left anterior middle temporal lobe and precentral gyrus. Finally, a moderately inclusive model was observed for fluency, where relatively higher coefficients were located near the premotor and thalamic regions. In contrast to the FC results, applying the filtering method to the SC made little difference as the models were generally sparse and visually interpretable using nodal degree. Interestingly, the nodal degree analysis for each behavioural factor identified nodes within (or around) the lesion correlates found in Fig. 3 (Fig. 5, middle column), where the nodes represented predominantly positive weights.

The squared-error model performance of the SC data was not significantly better than the lesion models for any behavioural factor (Table 1); in fact, for phonology it was significantly worse (*P* = 0.00046). We also did not identify any significant models when using the SC predictors to predict the residual factor scores (after accounting for T_1_ lesion).

#### Combined functional and structural connectivity

There were 39 patients with both FC and SC data. We found that model performance for combined data was mixed compared to separate models, whereby significant models were found for phonology (*r* = 0.26, *P* = 0.049), semantics (*r* = 0.44, *P* = 0.004) and fluency (*r* = 0.31, *P* = 0.034), but not for executive function (max *r* = −0.28, ns). These models did not improve over models trained on structural or functional network data alone, or models trained only on T_1_ lesions either via comparison of squared prediction error (Table 1) or when predicting residual factor scores (after accounting for T_1_ lesion).

### Functional connectivity map of lesion regions of interest

**Figure 6:**
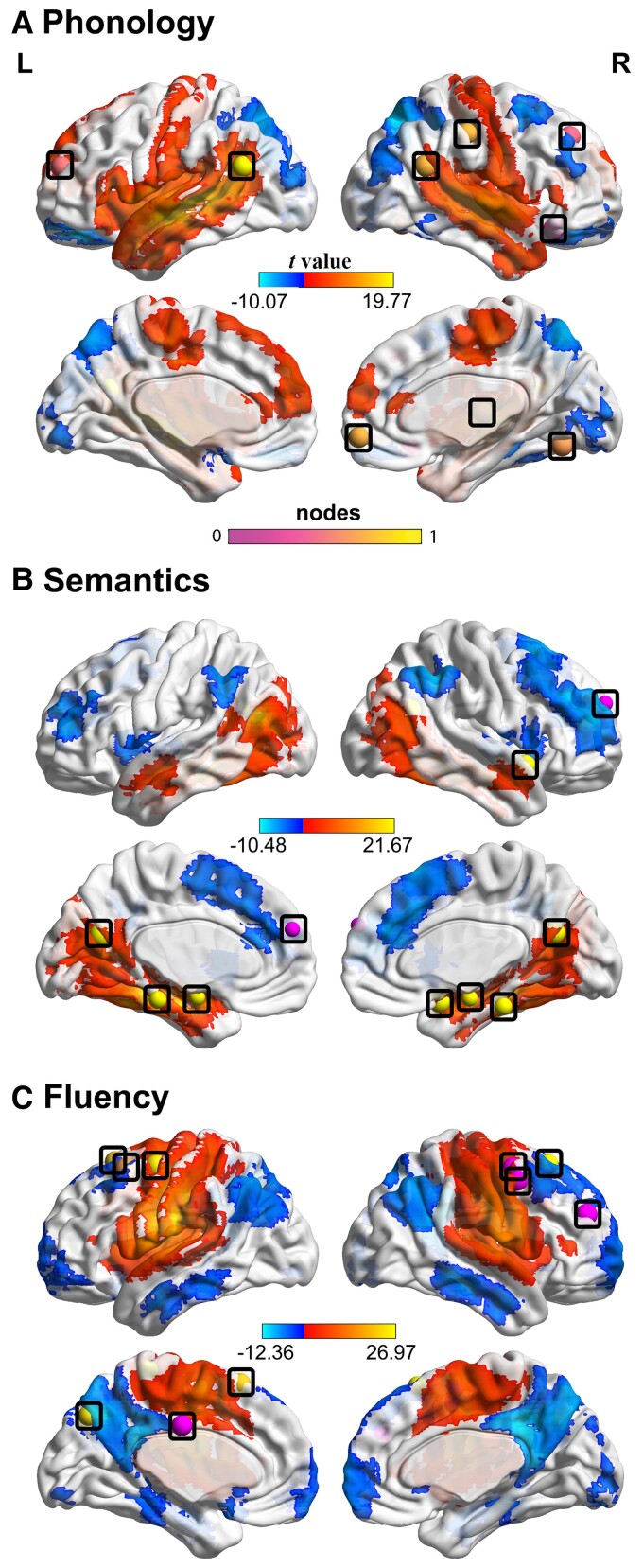
**Seed-based FC.** Healthy whole-brain FC maps using lesion correlates as seeds (restricted to grey matter) for (**A**) phonology, (**B**) semantics and (**C**) fluency. The scale denotes *t*-scores based on a one-sample *t*-test at the group level. Superimposed on each map are the most predictive nodes from the patient regularized regression analysis, nodes are indicated by percentage of positive connections.

Resting-state networks related to lesion correlates were identified within the healthy control group (Fig. 6) by inserting seed ROI based on the critical lesions for each behavioural factor (Supplementary material, section 7). The ROI related to phonology positively correlated with activity in bilateral temporal, motor and inferior frontal regions and negatively correlated with frontal and occipital regions. The ROI related to semantic ability positively correlated with bilateral temporal and occipital regions, particularly medial regions. Finally, the ROI related to fluency positively correlated with bilateral postcentral, precentral and supplementary motor areas. We used these healthy network maps to determine whether the nodes identified from the patient FC models overlapped (Fig. 6). Seven out of 10 nodes identified in the phonology model were found to be overlapping; 7 out of 10 nodes were observed in the semantic model and 9 out of 10 nodes for the fluency model. In summary, the FC maps generated in healthy subjects largely reflected the predictive nodes in patient FC models using the regularized regression models.

## Discussion

Neuroimaging data are increasingly being used to predict behavioural deficits, particularly post-stroke, to allow for a step change in how diagnosis, prognosis and rehabilitation are managed.^4,5,7,12–14,18,35,65,66^ Lesion-symptom mapping has been applied with success to account for some individual differences in performance and recovery potential following stroke. However, the severity and profile of impairment is not completely accounted for by characteristics of the lesion alone, which implies that the effects on brain function are not solely related to the lesion site. Complementary neuroimaging modalities such as SC and FC present a means of characterizing potential network-level disorder ensuing from focal lesions. Several studies suggest that individual differences in whole-brain connectivity profiles are predictive of symptom severity and profile post-stroke, yet it is unclear whether these network analyses account for variance that cannot be explained by the lesion alone. The present study investigated how well *in vivo* structural and functional networks map to aspects of language and cognition in chronic post-stroke aphasia patients using a range of regularized regression models, both before and after accounting for lesion effects. Overall, our results suggest that while FC and SC can predict behaviour, these models perform no better (nor account for additional residual variance) than lesion models informed by T_1_ imaging. In the following sections, we provide more context and discussion for each of the main themes.

### Utility of multimodal data

The lesion model converged with previous lesion-symptom mapping studies that have localized the orthogonal dimensions of stroke aphasia, including phonology (posterior portion of the superior temporal gyrus), semantic (anterior middle temporal lobe) and speech fluency/quanta (precentral gyrus) abilities,^8,34,36,38,39,42^ which in turn align with prominent models of language.^24,67,68^ In addition, our results indicate that pair-wise structural and/or functional connections do not improve predictions over and above lesion models.^12,13^ The result is unsurprising if the lesion and connectivity data are highly correlated as reported by Hope *et al.*,^12^ as this would result in redundant information/collinearity during modelling. The variability in results may be attributed to whether studies used *in vivo* or simulated disconnection measures, as a recent study showed that direct measures of FC were more predictive than simulated functional disconnections, however, structural disconnection often performed similarly to the former.^69^ However, failure to improve predictive accuracy using connectivity might be domain specific, as other longitudinal studies with *in vivo* connectivity data have shown benefit of FC for visual and verbal memory.^14^ In the current study, we found that SC nodes fell within the critical lesion region identified with T_1_ data alone—suggesting that the data are highly overlapping. In contrast to the SC models, the functional connections were much more distributed across both hemispheres (for phonology, semantics and fluency). Given the complex nature of functional networks, we explored how the patient models differed to functional networks in healthy subjects. Our results showed that most FC nodes identified in the patient population were located within healthy functional networks. In earlier work, these kinds of remote, functionally specific down-regulations of activity following focal lesions were termed examples of ‘functional’ or ‘dynamic’ diaschisis.^70^ More recent considerations of diaschisis^71^ have proposed that gradual normalization of function/activation is a core definitional feature of all diaschisis types and that any changes observed chronically must reflect post-damage neuroplasticity. In the context of network functional dynamics and distributed computational models, this definition could be debated given that remote changes could occur both immediately and persistently after damage. Given that we (like most studies of chronic patients) do not have measures of the same patients in the acute phase to differentiate these possibilities, henceforth we will refer to the observed changes with the agnostic term ‘functionally specific reorganization’. A direct example in this study is observed in the FC model for semantics, which revealed that almost all 10 strongest nodes were located in the healthy semantic network, with similar results for phonology and fluency. We suggest, therefore, that standalone models using structural or functional connections may reflect lesion triggered functionally specific reorganization in stroke aphasia. The implication of this result would be that lesion information obtained using relatively basic imaging might be sufficient to predict behavioural status, as opposed to collecting and analysing specialized diffusion and functional MRI. Overall, our findings suggest that accounting for lesion location is fundamental in understanding whether/how connectivity measures influence prediction models. Here, we suggest that functionally specific reorganization can provide an explanation of why previous studies have found significant FC models but we cannot rule out whether this is colinear with the lesion effect.

### Unpacking multivariate connectivity models

Mapping brain–behaviour relationships is challenging largely due to the vast number of features that can be extracted from neuroimaging. The consequence on univariate analysis is the increase of false positives; however, the multivariate case can be even more dire, as perfect model fits can be obtained for random data if the number of predictors is *n −* 1 or greater than the number of observations. This is often referred to as the dimensionality problem, which leads to overfitting the data. One simple way to apply constraints is to limit the number of features within the model space (e.g. Yourganov *et al*.^9^ used *a priori* hypotheses or feature selection method) with the caveat that one cannot draw conclusions beyond that specific feature space. Regularized regression offers an alternative solution that can be applied to every feature by imposing constraints on the degrees of freedom such that the models do not overfit the data. Specifically, Siegel *et al*.^14^ mapped functional connections to behavioural deficits post-stroke using ridge regression but limited the interpretation at a macro-scale (inter- or intra-hemispheric relationships). Ridge models spread weights among correlated variables such that the magnitude of the weight on any given variable no longer corresponds with its importance. In contrast, LASSO models seek the fewest number of uncorrelated variables, thus implicate an implausibly sparse selection of voxels that are difficult to situate within the regional and network architecture of the brain. Our results showed that (i) the best performing models are rarely placed in the extreme ends of this spectrum; in contrast, an elastic net approach is often preferred; and (ii) no single, fixed sparsity parameter was appropriate for all behavioural targets or across imaging modalities. These results indicate that sparsity should be optimized for each behavioural target.

Another way to simplify the model was to use nodal degree. This network-based metric has been commonly used to measure the importance of nodes by summing the number (or weights) of the node’s connections.^72,73^ We explicitly note that this method does not represent the true statistics of the model (as not all nodes are represented and a relatively arbitrary cut off was used). It does, however, provide a simplified model that can be scrutinized. For example, it becomes apparent that predictive nodes using SC sit within or around the core lesions identified in lesion models. In contrast, high degree nodes from the FC models in patients were shown to be overlapping with healthy FC networks. A secondary filtering step showed a reduced number of connections emanating from the top 10 nodes. When the original model was sparse, the filtering procedure did not greatly change the connectivity profile; however, when the model was dense (e.g. SC model for fluency) the filtering allowed us to observe that most predictive weights were within the left hemisphere surrounding the critical lesion. Further work will be required to explore the use of applying this form of filtering to dense models.

### Limitations

It is clear that there is a need to improve the accuracy of diagnosis and prognosis in stroke aphasia to help refine rehabilitation pathways and manage long-term care. In the current study, we used cross-sectional data to make predictions about current (chronic) state to help refine our understanding of how brain and behaviour are linked. There is an obvious issue with cross-sectional data, such that we cannot make prognostic predictions (in the temporal sense) and also importantly during the recovery process there are likely to be other important factors related to neural reorganization. There is still a dearth of longitudinal (acute to chronic) data in stroke aphasia to formally investigate prognosis using structural^16,35,74^ and/or functional (see recent meta-analysis^75^) neuroimaging. There are critical practical issues associated with collecting longitudinal data that limit our ability to explore prognosis: (i) samples tend to dominated by moderate to mild cases who continue to become mild or recovered in the chronic stage, whereas a typical cross-sectional chronic sample tends to span a broader range of severities^76,77^; (ii) the times at which patients are tested acutely vary, at least for functional imaging it is rare to find populations tested within 6 months^75^; (iii) behavioural testing is often limited in the variety and depth of data^78^; and (iv) the overall sample size in longitudinal studies is generally smaller than chronic-only studies. These issues highlight the importance of open science and international collaboration to build large and sensitive datasets to tackle these problems. An alternative approach to collecting multimodal neuroimaging data directly from patients is to create simulated data such as indirect disconnection mapping.^69^ These methods only require the location of damage/abnormality that can then be used to infer network disconnections based on healthy datasets; however, the accuracy of the methods require validation in independent samples that have both *in vivo* and simulated data.^79^

## Conclusion

This study used *in vivo* FC and SC measures to predict behavioural deficits post-stroke across a range of sparsities. Our results are contrary to recent studies that have suggested model improvements when combining multimodal data, but this may be related to whether lesion effects are accounted for in the first instance. It is important to note that this does not mean that connections are not important when considering neurocognitive mechanistic accounts of aphasia^80^ and recovery,^81^ but, in the context of building brain-to-behaviour prediction models, the lesion and disconnections are highly collinear and thus information about disconnections tends not to improve the behavioural predictions. This may well reflect the fact that, in stroke, the infarct induces full-depth, grey and white matter damage. This may not be the case for all disease types; for example, neuro-degenerative disorders can display atrophy at a slower rate and at the end of the pathology cascade. Accordingly, in these disorders, disconnections and other pathological markers are often the precursors rather than the result of structural abnormalities as detected using typical T_1_ data.^82,83^

## Supplementary Material

awac388_Supplementary_Data
