# Supplementary material for: Using in vivo functional and structural connectivity to predict chronic stroke aphasia deficits
Source: Brain. 2022 Nov 8;146(5):1950–62. doi: 10.1093/brain/awac388 (PMC10151190; doi:10.1093/brain/awac388)
Supplement: awac388_Supplementary_Data [file awac388_supplementary_data.pdf]

Supplementary Materials 1

Neuropsychological assessments

The battery included a subset of tasks from the Psycholinguistic Assessments of Language Processing in Aphasia (PALPA) battery[1]: (1) auditory discrimination using non-word minimal pairs, (2) auditory discrimination using word minimal pairs, (3) immediate repetition of non-words, (4) immediate repetition of words, (5) delayed repetition of non-words, and (6) delayed repetition of words. Tasks from the 64-item Cambridge Semantic Battery[2] were included: (7) word-to-picture matching task (spoken version), (8) word-to-picture matching task (written version), (9) Camel and Cactus Test (picture), and (10) picture naming test. Other language tasks included (11) the Boston Naming Test (BNT)[3], (12) written 96-trial synonym judgement test[4], (13) the spoken sentence comprehension task from the Comprehensive Aphasia Test (CAT)[5], and the 'Cookie theft' picture description task from the Boston Diagnostic Aphasia Examination[6]. Specifically, patients' responses in the 'Cookie theft' picture description task were recorded and transcribed. The (14) number of word tokens (T), (15) type/token ratio (TTR), (16) mean length of utterance in morphemes (MLU), and (17) words-per-minute (WPM) were computed. Cognitively demanding tasks include (18) forward and (19) backward digit span[7], (20) the Brixton Spatial Rule Anticipation Task[8], and (21) Raven's Coloured Progressive Matrices[9].

Supplementary Materials 2

Acquisition of neuroimaging data and pre-processing

All patients had high resolution structural T1-weighted MRI scans, which were acquired on a 3.0 Tesla Philips Achieva scanner (Philips Healthcare, Best, The Netherlands) using an 8-element SENSE head coil. A T1-weighted inversion recovery sequence with 3D acquisition was employed, with the following parameters: TR (repetition time) = 9.0 ms, TE (echo time) = 3.93 ms, flip angle = 8°, 150 contiguous slices, slice thickness = 1 mm, acquired voxel size $1.0 \times 1.0 \times 1.0\ mm^3$, matrix size 256 × 256, FOV = 256 mm, TI (inversion time) = 1150 ms, and SENSE acceleration factor 2.5. The total scan acquisition time was 575 s.

We acquired rs-fMRI on a subset of patients (N = 39) and younger healthy controls N = 30). We used a dual gradient echo planar imaging technique for the rs-fMRI protocol in order to improve signal detection within inferior temporal and orbitofrontal regions[10–12]. We used the following parameters: TEs = 12 and 35 ms, TR = 2.8 s, voxel size = 3 × 3 × 4 mm, FOV = 240

× 124 × 240 mm, matrix size = 80 × 80, flip angle = 85°. A total of 130 volumes were collected over 6.25 min. Participants were instructed to look at a fixation cross and lie still during scanning.

Diffusion-weighted images were acquired using a pulsed gradient spin echo (PGSE) echo planar imaging (EPI) sequence implemented with TE = 54ms, Gmax = 62 mT/m, half scan factor = 0.679, 112 x 112 image matrix reconstructed to 128 x 128 using zero filling, reconstructed in-plane resolution 1.875 x 1.875 mm^2, slice thickness 2.1 mm, 60 contiguous slices, 43 non-collinear diffusion sensitisation directions at b = 1200 s/mm2 (Δ = 29.8ms, δ = 13.1ms), 1 at b = 0, SENSE acceleration factor 2.5 and with a variable TR due to cardiac gating. A peripheral pulse unit was placed on the participant's index finger to measure the cardiac cycle and aimed at reducing artefacts associated with pulsatile brain movements[13]. The total scan time was approximately 28 minutes. Each diffusion-weighted volume was acquired entirely before starting on the next diffusion weighting, resulting in 44 temporally spaced volumes with different gradient directions. For each run, phase encoding was performed in right-left and left-right directions, giving two sets of images with the same diffusion gradient directions but opposite polarity k-space traversal, and hence reversed phase and frequency encoded direction[14].

High-resolution structural scans were pre-processed with the same procedure as our previous studies[15,16] using Statistical Parametric Mapping software (SPM8: Wellcome Trust Centre for Neuroimaging, http://www.fil.ion.ucl.ac.uk/spm/) and a modified segmentation-normalisation procedure[17]. After normalising individual lesioned brain images into standard Montreal Neurological Institute (MNI) space, images were smoothed with an 8mm full-width-half-maximum (FWHM) Gaussian kernel. Lesions were automatically identified for each patient by comparing the structural image with an age and education matched control group, using an outlier detection algorithm to identify 'abnormal' voxels[17]. All parameters were kept at default except the lesion definition 'U-threshold', which was set 0.5 after comparing the results obtained from a sample of patients to what would be nominated as lesioned tissue by an expert neurologist. All resultant lesion maps were visually inspected and any discrepancies were manually corrected. The overall lesion maps were concatenated across the patients in the current study and shown in Figure 1.

The resting-state data were processed using SPM8 and the Data Processing Assistant for Resting State fMRI (DPARSF Advanced Edition, version 2.3) toolbox[18]. The pre-processing pipeline for the functional data included: discarding the first two time-points for signal stabilisation, slice time correction, volume realignment, dual-echo combination (linear average), and co-registration to the T1. All functional data were then inspected using an artefact rejection tool (ART; http://www.nitrc.org/projects/artifact_detect/) in order to identify time-points with high motion and/or signal artefacts. Volumes were censored as outliers if they had an intensity ± 2.5 standard deviations from the mean intensity and/or had greater than 1 mm movement in any direction. In addition to regressing out outlier volumes, we added 24 movement parameters as regressors in order to account for movement related artefacts[19]. We used DPARSFA to regress out the influence of nuisance variables, including global signal, white matter signal, mean CSF signal, and the 24 movement parameters so that the effects of head movements could be better controlled[20,21]. The images were normalized using the native-to-MNI transformation matrix obtained from the modified normalisation procedure by Seghier and colleagues[17] and then smoothed using 8 mm FWHM Gaussian kernel. Finally, we removed a linear trend and filtered the time series between 0.01 ~ 0.08 Hz.

Processing of the DTI data was conducted using FSL's (v5.0.10) diffusion pipeline[22]. Firstly, a brain extraction was performed on the B0 image using the brain extraction tool (BET)[23]. The data were prepared and submitted to FSL's TOPUP tool in order to estimate and correct susceptibility inducted distortions[24], where the off-resonance field is estimated and then the two images are combined into a single corrected one. The eddy tool was used to correct for distortions such as eddy currents and head motion[25] and we used the replace outliers flag[26]. Bayesian Estimation of Diffusion Parameters Obtained using Sampling Techniques (BEDPOSTX) was then used to run Markov Chain Monte Carlo sampling to build up distributions on diffusion orientations at each voxel[27,28] using the following parameters; number of fibres per voxel = 3, model = 3 (deconvolution model with zeppelin), burn-in period = 3000, number of jumps = 1250.

Supplementary Materials 3

Table. Detailed demographic and neuropsychological profile for all subjects.

ID	Sex	Age (years)	Education (years)	Months Post Stroke	BDAE	Lesion Vol (voxels 1mm3)	NonWord Repetition (immediate)	NonWord Repetition (delayed)	Word Repetition (immediate)	Word Repetition (delayed)	Words-to-picture matching (spoken)	Words-to-picture matching (written)	Cambridge Naming Test	Boston Naming Test	Camel and Cactus (pictures)	Synonym Judgement	CAT sentence comp-rehension	Ravens	Brixton Spatial Anti-cipation Test	Digit span (Forward)	Digit span (Backward)	Words-per-minute	Type-token ratio	Mean length per utterance	Tokens
1	M	66	11	126	Anomia	15492	83.33	92.59	100.00	98.75	100.00	100.00	83.87	66.66	82.54	84.94	100.00	94.44	83.30	50.00	57.14	51.52	67.50	58.30	25.40
2	F	77	16	34	Wernicke/Conduction	6843	13.33	3.70	45.00	41.25	96.88	100.00	20.96	12.28	85.72	81.72	50.00	80.56	45.62	25.00	28.57	31.95	52.71	64.44	64.44
4	M	76	11	116	TMA	11239	56.67	37.03	88.75	91.25	100.00	100.00	87.10	77.19	90.47	96.77	87.50	72.22	79.34	50.00	28.57	22.47	86.96	68.77	7.30
5	M	65	17	25	Anomia	4806	83.33	59.26	100.00	100.00	98.44	100.00	83.87	70.18	92.07	97.85	71.88	100.00	87.27	62.50	57.14	51.33	58.40	67.75	39.68
6	M	85	10	46	Broca	11393	0.00	0.00	3.75	0.00	95.31	92.19	1.61	0.00	90.47	84.94	84.38	80.56	55.54	50.00	42.86	1.30	75.00	8.49	1.27
7	M	63	12	24	TSA	5822	73.33	88.89	93.75	95.00	71.88	67.19	32.26	14.03	69.84	87.10	84.38	86.11	57.52	100.00	57.14	45.90	75.31	62.26	25.71
8	M	62	11	104	Broca	27242	3.33	0.00	5.00	1.25	92.19	100.00	3.23	1.76	85.72	80.65	31.25	91.67	41.65	0.00	0.00	34.24	55.17	33.11	9.21
9	M	52	17	33	Broca	11915	60.00	11.11	73.75	68.75	98.44	98.44	77.42	75.44	100.00	98.92	56.25	91.67	55.54	37.50	0.00	15.38	73.68	34.93	12.06
10	M	66	11	12	Global	14890	13.33	3.70	27.50	0.00	67.19	53.13	1.61	1.76	69.84	59.14	28.13	61.11	35.71	0.00	0.00	9.44	40.00	12.73	3.17
11	F	52	12	76	Anomia	9767	90.00	100.00	100.00	98.75	100.00	100.00	96.77	92.98	80.95	96.77	84.38	91.67	65.45	87.50	85.71	100.00	75.00	60.28	19.05
12	M	72	11	42	Global	27054	0.00	0.00	0.00	0.00	78.13	90.63	4.84	0.00	74.60	75.27	25.00	66.67	43.64	0.00	0.00	9.84	81.82	24.20	10.48
14	M	76	11	192	Mixed Nonfluent	42568	30.00	3.70	75.00	65.00	92.19	98.44	64.51	49.13	77.77	83.87	59.38	83.33	73.39	50.00	28.57	20.07	64.71	33.96	10.79
15	M	68	11	14	Mixed Nonfluent	8788	86.67	88.89	100.00	96.25	98.44	93.75	98.38	70.18	74.60	86.02	78.13	66.67	73.39	62.50	57.14	23.93	68.42	41.60	12.06
16	M	72	11	155	Global	32981	0.00	0.00	0.00	0.00	87.50	60.94	0.00	0.00	44.44	53.76	34.38	38.89	25.79	0.00	0.00	0.00	0.00	0.00	0.00
17	M	81	11	69	Mixed Nonfluent	28144	36.67	33.33	55.00	41.25	92.19	75.00	40.32	26.32	52.38	63.44	50.00	61.11	47.61	37.50	28.57	3.54	57.14	15.28	4.44
18	M	63	12	12	Anomia	18639	93.33	92.59	98.75	92.50	98.44	100.00	98.38	100.00	71.42	91.40	68.75	50.00	57.52	62.50	28.57	24.91	66.67	56.03	16.19
19	M	63	12	42	Mixed Nonfluent	31599	70.00	33.33	85.00	83.75	64.06	76.56	8.06	8.77	84.12	61.30	31.25	86.11	43.64	37.50	28.57	11.33	33.33	44.95	38.10
20	M	50	12	16	Broca	26218	93.33	70.37	100.00	81.25	96.88	95.31	70.96	33.34	74.60	77.42	65.63	91.67	63.47	37.50	28.57	6.01	48.57	20.61	22.22
21	M	58	11	135	Broca	18392	73.33	92.59	77.50	87.50	100.00	100.00	90.32	82.45	76.19	92.47	87.50	75.00	61.48	100.00	100.00	8.50	70.00	37.69	9.52
22	M	87	12	35	Anomia	8238	36.67	25.92	82.50	73.75	98.44	98.44	88.71	66.66	53.97	96.77	56.25	41.67	47.61	62.50	42.86	27.32	74.07	56.88	17.14
23	M	51	12	34	Broca	20043	33.33	3.70	56.25	26.25	93.75	95.31	43.55	15.79	85.72	83.87	46.88	100.00	83.30	0.00	0.00	9.60	54.10	34.38	19.37
24	M	58	13	32	Mixed Nonfluent	14625	0.00	0.00	6.25	0.00	92.19	98.44	4.84	1.76	79.37	70.97	28.13	88.89	67.44	0.00	0.00	7.73	38.89	14.55	5.71
25	F	67	14	176	Mixed Nonfluent	26283	53.33	40.74	70.00	81.25	95.31	93.75	74.19	68.42	92.07	87.10	56.25	38.89	45.62	25.00	0.00	30.25	71.43	65.37	20.00
26	M	75	11	12	Anomia	1481	63.33	40.74	86.25	81.25	93.75	90.63	77.42	66.66	79.37	77.42	84.38	86.11	65.45	62.50	28.57	26.80	80.00	32.75	11.11
27	F	48	12	16	Broca	5273	40.00	62.97	72.50	68.75	100.00	98.44	69.35	45.61	96.82	94.63	93.75	100.00	89.26	62.50	57.14	9.65	80.65	48.39	9.84
28	M	80	12	65	Broca	18163	36.67	70.37	90.00	91.25	98.44	98.44	74.19	56.14	84.12	78.49	75.00	77.78	47.61	62.50	42.86	15.56	78.57	42.02	8.89
29	M	67	11	60	Anomia	10073	56.67	55.56	82.50	88.75	96.88	100.00	83.87	82.45	88.89	95.70	90.63	88.89	83.30	87.50	57.14	43.02	64.63	36.38	26.03
30	M	67	11	120	Mixed Nonfluent	26097	36.67	44.44	85.00	78.75	100.00	98.44	82.26	66.66	88.89	65.59	78.13	80.56	67.44	37.50	0.00	30.10	64.71	41.77	10.79
31	M	68	12	50	Global	41379	0.00	0.00	37.50	0.00	57.81	31.25	0.00	0.00	53.97	50.54	12.50	30.56	41.65	25.00	0.00	41.21	46.88	41.77	10.16
32	M	44	11	40	Anomia	8437	100.00	100.00	100.00	100.00	100.00	100.00	91.93	57.89	92.06	93.55	87.50	97.22	75.37	50.00	57.14	26.44	66.07	75.39	17.78
33	M	61	11	16	Broca	3528	23.33	18.52	48.75	32.50	100.00	100.00	43.55	15.79	92.06	91.40	87.50	83.33	71.40	62.50	42.86	7.87	80.00	14.82	7.94
34	M	73	11	23	Mixed Nonfluent	22732	26.67	18.52	50.00	61.25	96.88	93.75	48.38	40.35	60.32	59.14	12.50	38.89	63.47	37.50	28.57	3.50	18.18	16.21	10.48
35	F	53	11	47	Anomia	1526	66.67	40.74	81.25	78.75	100.00	98.44	85.48	40.35	92.06	86.02	75.00	66.67	51.57	50.00	28.57	26.89	67.86	75.39	17.78
36	F	51	11	66	Anomia	6975	53.33	11.11	93.75	41.25	100.00	96.88	90.32	52.63	93.65	80.65	62.50	88.89	47.60	37.50	28.57	23.36	72.34	50.94	14.92
37	M	54	13	35	Broca	18632	0.00	0.00	0.00	0.00	100.00	98.44	4.84	0.00	84.12	80.65	75.00	88.89	83.31	37.50	42.86	49.84	47.95	96.78	46.35
38	F	77	11	56	Anomia	13577	23.33	14.81	57.50	55.00	96.88	98.44	54.84	52.63	87.30	84.95	68.75	63.89	33.72	75.00	42.86	12.01	60.00	20.38	7.94
39	F	52	11	99	Mixed Nonfluent	40313	33.33	0.00	70.00	18.75	89.06	96.88	45.16	21.05	85.71	79.57	68.75	91.67	55.54	25.00	0.00	3.85	100.00	8.91	3.49
40	F	69	19	39	Anomia	9159	50.00	51.85	90.00	88.75	100.00	100.00	91.93	45.61	96.82	100.00	81.25	100.00	71.40	50.00	57.14	50.14	46.98	100.00	100.00
41	M	78	13	36	Mixed Nonfluent	34242	23.33	7.41	43.75	35.00	100.00	96.88	40.32	10.53	74.60	67.74	34.38	77.78	65.45	75.00	42.86	2.43	90.91	7.13	3.49
42	M	68	11	21	Anomia	3311	83.33	77.78	96.25	96.25	100.00	100.00	87.09	64.91	85.71	81.72	78.13	77.78	71.40	87.50	42.86	43.68	72.97	66.22	23.49
43	F	68	16	22	Anomia	8118	90.00	88.89	97.50	95.00	100.00	100.00	96.77	89.47	95.24	96.77	96.88	97.22	73.39	75.00	71.43	46.51	70.11	87.44	27.62
44	M	59	13	37	Broca	13080	10.00	3.70	51.25	42.50	98.44	100.00	56.45	24.56	93.65	84.95	56.25	88.89	47.60	25.00	28.57	13.94	67.74	34.81	9.84
45	M	59	11	34	Anomia	16433	43.33	51.85	95.00	95.00	98.44	100.00	74.19	59.65	95.24	92.47	78.13	97.22	85.29	37.50	28.57	26.63	52.13	60.56	29.84
46	M	58	13	57	Global	33239	0.00	0.00	1.25	0.00	78.13	93.75	0.00	0.00	68.25	77.42	46.88	91.67	100.00	25.00	0.00	0.00	0.00	0.00	0.00
47	M	51	13	72	Anomia	22948	26.67	14.81	86.25	63.75	95.31	98.44	79.03	43.86	80.95	77.42	75.00	88.89	96.97	37.50	28.57	26.59	51.64	84.33	38.73
48	F	46	16	21	Conduction	3897	0.00	0.00	38.75	23.75	100.00	100.00	35.48	15.79	93.65	92.47	71.88	97.22	87.27	37.50	0.00	12.97	55.26	16.07	12.06
49	M	82	10	13	Broca	12131	33.33	18.52	65.00	66.25	96.88	98.44	67.74	40.35	82.54	88.17	90.63	88.89	67.44	87.50	28.57	8.10	83.33	40.75	5.71
50	M	79	11	64	Global	23860	0.00	0.00	0.00	0.00	32.81	28.13	0.00	0.00	34.92	49.46	25.00	47.22	35.70	25.00	0.00	0.00	0.00	0.00	0.00
51	M	68	11	37	Conduction	4773	0.00	3.70	35.00	22.50	100.00	100.00	16.13	8.77	85.71	74.19	87.50	69.44	59.50	37.50	42.86	20.78	60.61	48.39	20.95
52	F	44	13	37	Anomia	18948	60.00	44.44	92.50	88.75	100.00	100.00	87.09	63.16	92.06	90.32	84.38	91.67	83.31	50.00	28.57	11.22	60.53	34.81	12.06
53	F	73	11	46	TMA	23863	56.67	48.15	85.00	91.25	100.00	100.00	62.90	40.35	85.71	86.02	87.50	80.56	55.54	75.00	0.00	9.32	64.00	24.01	7.94
54	F	75	11	160	Mixed Nonfluent	12057	0.00	0.00	0.00	0.00	100.00	95.31	4.84	5.26	79.36	76.34	56.25	50.00	37.69	25.00	0.00	7.24	39.13	26.60	14.60
55	M	84	9	35	Anomia	7854	13.33	18.52	61.25	53.75	96.88	98.44	87.09	66.67	96.82	96.77	62.50	80.56	79.34	62.50	57.14	28.33	77.50	42.45	12.70
56	M	74	11	18	Global	19500	0.00	0.00	36.25	0.00	76.56	59.38	6.45	1.75	61.90	44.09	50.00	47.22	51.57	25.00	0.00	8.50	33.33	10.19	1.90
58	F	43	16	15	Anomia	175	86.67	88.89	100.00	100.00	98.44	98.44	96.77	92.98	85.71	89.25	90.63	94.44	77.36	62.50	57.14	32.81	90.91	52.64	6.98
59	M	64	11	29	Mixed Nonfluent	33239	53.33	22.22	88.75	38.75	62.50	92.19	14.52	3.51	66.66	48.39	46.88	83.33	71.40	37.50	0.00	15.50	29.31	26.32	18.41
60	M	67	11	44	Mixed Nonfluent	31317	0.00	0.00	5.00	0.00	78.13	56.25	1.61	0.00	60.32	50.54	50.00	63.89	37.69	25.00	0.00	0.47	100.00	0.85	0.32
61	M	79	11	63	Mixed Nonfluent	33678	16.67	3.70	62.50	32.50	85.94	60.94	25.81	17.54	53.97	47.31	43.75	61.11	37.69	25.00	0.00	3.54	66.67	24.20	3.81
62	M	45	11	25	Anomia	10409	70.00	74.07	87.50	72.50	100.00	100.00	90.32	77.19	92.06	90.32	93.75	97.22	83.31	50.00	28.57	20.92	89.58	73.86	15.24
63	F	58	11	278	Anomia	12699	60.00	62.96	95.00	93.75	98.44	98.44	87.09	80.70	84.12	91.40	81.25	83.33	69.42	62.50	0.00	17.61	86.96	23.77	7.30
64	M	67	11	13	Conduction	4879	0.00	3.70	13.75	7.50	100.00	98.44	6.45	5.26	90.47	72.04	59.38	80.56	81.32	25.00	28.57	51.94	65.45	47.30	17.46
65	M	52	11	73	Global	37822	0.00	0.00	0.00	0.00	59.38	65.63	0.00	0.00	63.49	53.76	34.38	88.89	57.52	0.00	0.00	0.00	0.00	0.00	0.00
66	M	86	9	17	Anomia	8528	30.00	11.11	76.25	71.25	98.44	98.44	56.45	57.89	76.19	97.85	75.00	52.78	77.36	62.50	42.86	46.09	56.56	86.60	38.73
67	M	73	11	114	Broca	36877	10.00	14.81	65.00	55.00	92.19	98.44	61.29	54.39	66.66	72.04	62.50	47.22	33.72	25.00	28.57	9.28	63.16	16.74	6.03
68	M	67	17	14	Conduction	6557	3.33	3.70	21.25	5.00	96.88	96.88	3.23	1.75	90.47	92.47	62.50	83.33	67.44	25.00	28.57	44.59	50.74	96.78	64.44
69	M	56	16	17	Anomia	6974	80.00	51.85	91.25	98.75	95.31	96.88	90.32	82.46	93.65	98.92	93.75	83.33	75.37	50.00	42.86	22.51	55.17	85.32	36.83
70	M	65	10	85	Anomia	6607	46.67	33.33	71.25	67.50	100.00	100.00	80.64	57.89	88.89	90.32	84.38	91.67	77.36	62.5	42.86	26.07	59.42	58.94	21.90
71	M	50	19	16	Anomia	4538	50.00	40.74	85.00	90.00	100.00	100.00	80.64	80.70	93.65	98.92	100.00	94.44	81.32	62.5	42.86	23.36	51.06	69.40	29.84
3	M	85	10	69	Anomia	21489	36.67	25.92	83.75	80.00	93.75	93.75	67.74	68.42	87.30	88.18	37.50	75.00	27.76	50.00	42.86	16.53	79.37	62.82	20.00
13	F	73	11	89	Anomia	8921	93.33	74.08	96.25	98.75	100.00	100.00	100.00	84.21	95.24	94.63	71.88	83.33	55.54	62.50	57.14	32.89	60.00	50.30	20.63

Supplementary Materials 4

Table. Loadings of behavioural assessments on components extracted from the rotated principal component analysis.

Tasks	Component			
	**Phonology**	**Semantics**	**Executive**	**Fluency**
Delayed Repetition - Words	**0.888**	0.221	0.183	0.193
Delayed Repetition - Non-words	**0.883**	0.027	0.237	0.148
Immediate Repetition - Non-words	**0.881**	0.061	0.231	0.143
Immediate Repetition - Words	**0.858**	0.211	0.125	0.170
Boston Naming Test	**0.823**	0.381	0.077	0.121
Cambridge Naming Test	**0.813**	0.431	0.154	0.117
Forward Digit Span	**0.746**	0.233	0.188	0.073
Backward Digit Span	**0.595**	0.207	0.234	0.359
CAT Spoken Sentence Comprehension	**0.521**	0.455	0.441	0.163
Spoken Word to Picture Matching	0.236	**0.801**	0.267	0.145
Type/Token ratio	0.362	**0.718**	-0.075	-0.092
Written Word to Picture Matching	0.182	**0.713**	**0.504**	0.155
Camel and Cactus Test: Pictures	0.092	**0.688**	0.484	0.288
96 Synonym Judgement	0.381	**0.658**	0.315	0.359
Minimal Pairs - Non-words	0.353	0.058	**0.814**	-0.014
Raven's Coloured Progressive Matrices	0.048	0.274	**0.735**	0.156
Minimal Pairs - Words	0.419	0.168	**0.705**	0.132
Brixton Spatial Anticipation Test	0.132	0.178	**0.698**	0.231
Token	0.010	0.034	0.207	**0.885**
Mean Length of Utterance in Morphemes	0.314	0.252	0.137	**0.831**
Words-Per-Minute	0.314	0.096	0.080	**0.768**

Factor loadings > 0.5 are given in bold; CAT = Comprehensive Aphasia Test.

Supplementary Materials 5

T1 predicting behaviours in regularised regression models

For completeness, we also re-analysed the T1 data using the same multivariate modelling process. The T1 model alpha distributions are relatively even. The T1 prediction models are significant for phonology(r = 0.52, p = 0.001), semantics (r = 0.40, p = 0.001), executive function (r = 0.36, p = 0.001), and fluency (r = 0.30, p = 0.005).

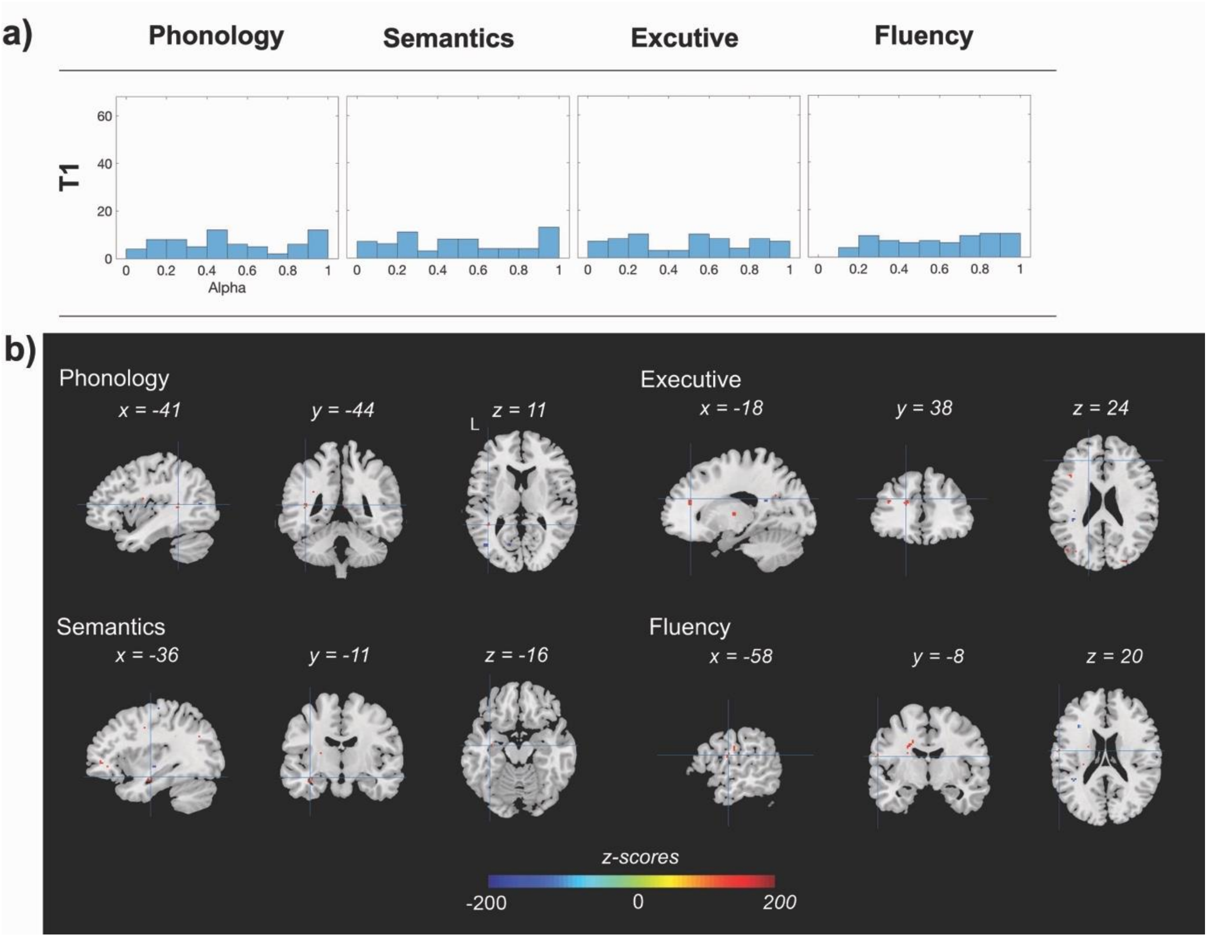

Figure. Mapping lesion to behavioural factor scores using multivariate regularise regression models (optimising alpha using nested cross validation). 5a shows the distribution of optimal alpha (i.e., 0 is dense and 1 is sparse) from each training fold. 5b shows the neural projections of the beta weights from the regularised model averaged across all training folds.

Supplementary Materials 6

Alpha values distribution when optimised in training sets

Since alpha was trained in the training set, each fold of training set had its own optimal alpha value. In the final model, the optimal alpha is not a single best value but a distribution of alpha values produced from each training set. The distribution is not even. However, there is a most favoured range in some domains and modalities. For example, the phonology, the functional connectivity model indicates a ridge model while the structural connectivity indicates a lasso model. The most frequently chosen alpha (based the training set) range is close to the post hoc alpha that we presented in the main manuscript.

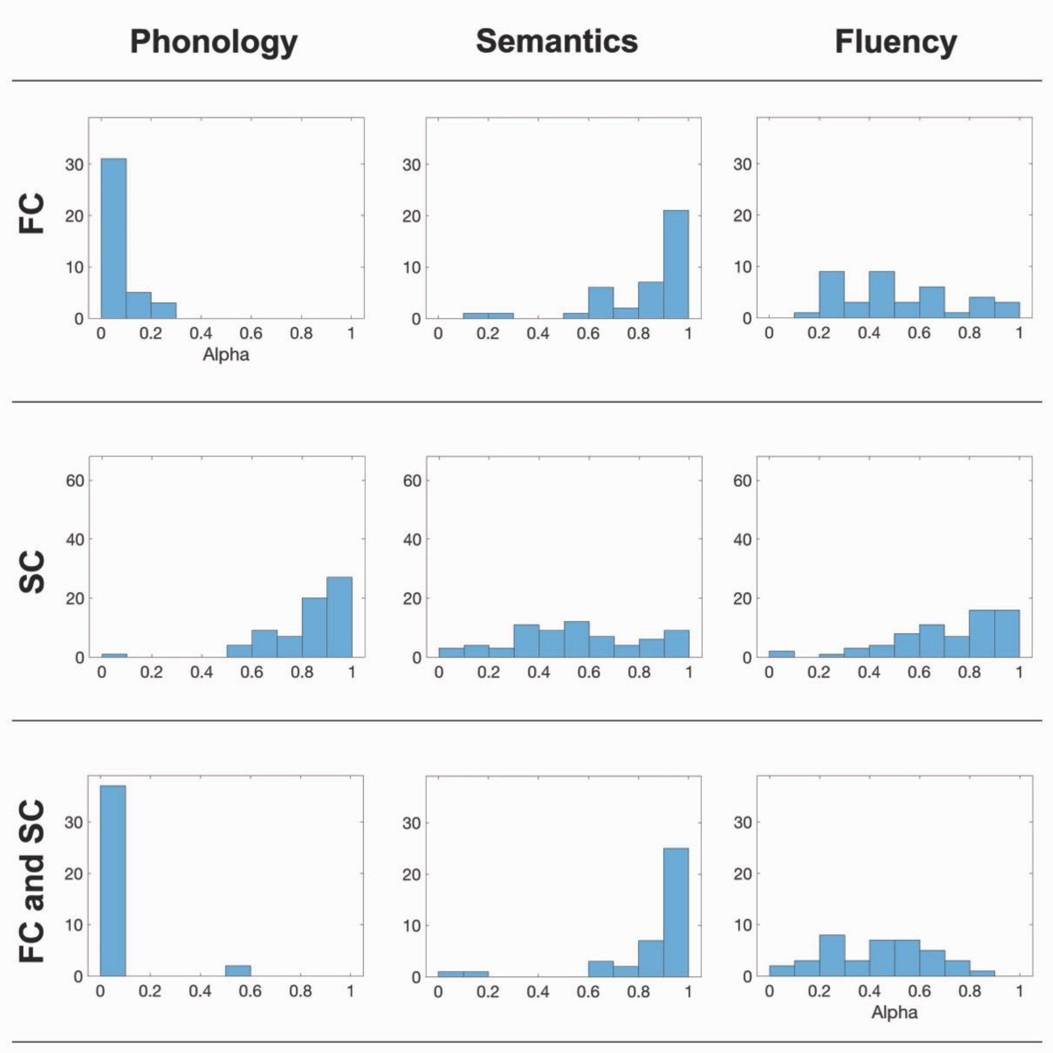

Figure. Alpha values distribution when optimised in training set. Optimise alpha in each fold might be different in each training fold and the distribution is not even for each behaviour in each modality.

Supplementary Materials 7

Functional connectivity of lesion predictors

Lesion correlates for four behavioural factor scores in the stroke aphasic population (Supplementary Materials 7 Figure). The identified clusters were used as seeds in the healthy rs-fMRI dataset in order to identify typical functional connectivity networks associated with these critical regions per behaviour (i.e., phonology, semantics, speech fluency and executive function).

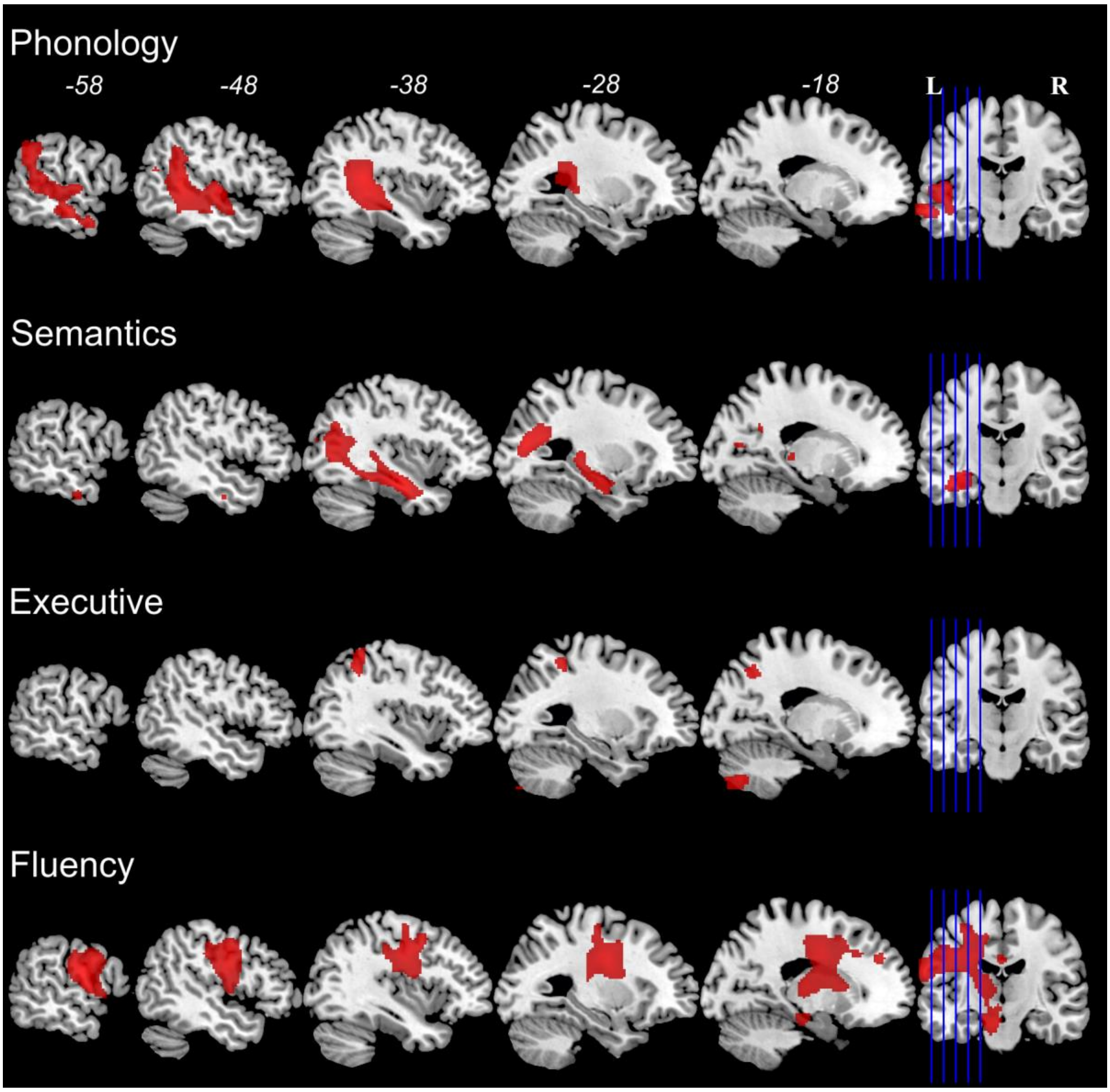

Figure. The significant regions using the whole patient group (68 patients). The threshold was voxel FDR corrected $p < 0.05$ and cluster > 2 cm^3. The ROIs in phonology, semantics, and fluency were used as seed ROIs in the FC analysis of the healthy control group.

References

1. Kay J, Lesser R, Coltheart M. *Psycholinguistic Assessments of Language Processing in Apshasia: PALPA*. 1st ed. Psychology Press; 1992.
2. Bozeat S, Lambon Ralph MA, Patterson K, Garrard P, Hodges JR. Non-verbal semantic impairment in semantic dementia. *Neuropsychologia*. 2000;38(9):1207-1215. doi:10.1016/S0028-3932(00)00034-8
3. Kaplan E, Goodglas H, Weintraub S. *The Boston Naming Test*. 2nd ed. Lea & Febinger; 1983.
4. Jefferies E, Patterson K, Jones RW, Lambon Ralph MA. Comprehension of Concrete and Abstract Words in Semantic Dementia. *Neuropsychology*. 2009;23(4):492-499. doi:10.1037/a0015452
5. Swinburn K, Baker G, Howard D. *CAT: The Comprehensive Aphasia Test*. Psychology Press; 2005.
6. Goodglass H, Kaplan E. *The Assessment of Aphasia and Related Disorders: Revised*. Lea & Febiger; 1972.
7. Wechsler DA. *Wechsler Memory Scale - Revised*. Psychological Corporation; 1987.
8. Burgess PW, Shallice T. *The Hayling and Brixton Tests*. Pearson Clinical; 1997.
9. Raven JC. *Advanced Progressive Matrices, Set II*. H. K. Lewis; 1962.
10. Poser BA, Versluis MJ, Hoogduin JM, Norris DG. BOLD contrast sensitivity enhancement and artifact reduction with multiecho EPI: Parallel-acquired inhomogeneity-desensitized fMRI. *Magn Reson Med*. 2006;55(6):1227-1235. doi:10.1002/mrm.20900
11. Halai AD, Welbourne SR, Embleton K, Parkes LM. A comparison of dual gradient-echo and spin-echo fMRI of the inferior temporal lobe. *Hum Brain Mapp*. 2014;35(8):4118-4128. doi:10.1002/hbm.22463
12. Halai AD, Parkes LM, Welbourne SR. Dual-echo fMRI can detect activations in inferior temporal lobe during intelligible speech comprehension. *Neuroimage*. 2015;122:214-221. doi:10.1016/j.neuroimage.2015.05.067
13. Jones DK, Pierpaoli C. Contribution of cardiac pulsation to variability of tractography results. In: *Proceedings of the International Society for Magnetic Resonance in Medicine*. ; 2005:222.
14. Embleton K V., Haroon HA, Morris DM, Ralph MAL, Parker GJM. Distortion

correction for diffusion-weighted MRI tractography and fMRI in the temporal lobes. *Hum Brain Mapp*. 2010;31(10):1570-1587. doi:10.1002/hbm.20959

15. Butler RA, Lambon Ralph MA, Woollams AM. Capturing multidimensionality in stroke aphasia: Mapping principal behavioural components to neural structures. *Brain*. 2014;137(12):3248-2366. doi:10.1093/brain/awu286
16. Halai AD, Woollams AM, Lambon Ralph MA. Using principal component analysis to capture individual differences within a unified neuropsychological model of chronic post-stroke aphasia: Revealing the unique neural correlates of speech fluency, phonology and semantics. *Cortex*. 2017;86:275-289. doi:10.1016/j.cortex.2016.04.016
17. Seghier ML, Ramlackhansingh A, Crinion J, Leff AP, Price CJ. Lesion identification using unified segmentation-normalisation models and fuzzy clustering. *Neuroimage*. 2008;41(4):1253-1266. doi:10.1016/j.neuroimage.2008.03.028
18. Chao-Gan Y, Yu-Feng Z. DPARSF: A MATLAB toolbox for “pipeline” data analysis of resting-state fMRI. *Front Syst Neurosci*. 2010;4:00013. doi:10.3389/fnsys.2010.00013
19. Friston KJ, Williams S, Howard R, Frackowiak RSJ, Turner R. Movement-related effects in fMRI time-series. *Magn Reson Med*. 1996;35(3):346-355. doi:10.1002/mrm.1910350312
20. Yan CG, Cheung B, Kelly C, et al. A comprehensive assessment of regional variation in the impact of head micromovements on functional connectomics. *Neuroimage*. 2013;76:183-201. doi:10.1016/j.neuroimage.2013.03.004
21. Power JD, Schlaggar BL, Petersen SE. Recent progress and outstanding issues in motion correction in resting state fMRI. *Neuroimage*. 2015;105:536-551. doi:10.1016/j.neuroimage.2014.10.044
22. Smith SM, Jenkinson M, Woolrich MW, et al. Advances in functional and structural MR image analysis and implementation as FSL. *Neuroimage*. 2004;23:S208-219. doi:10.1016/j.neuroimage.2004.07.051
23. Smith SM. Fast robust automated brain extraction. *Hum Brain Mapp*. 2002;17(3):143-155. doi:10.1002/hbm.10062
24. Andersson JLR, Skare S, Ashburner J. How to correct susceptibility distortions in spin-echo echo-planar images: Application to diffusion tensor imaging. *Neuroimage*. 2003;20(2):870-888. doi:10.1016/S1053-8119(03)00336-7
25. Andersson JLR, Sotiropoulos SN. An integrated approach to correction for off-resonance effects and subject movement in diffusion MR imaging. *Neuroimage*.

2016;125:1063-1078. doi:10.1016/j.neuroimage.2015.10.019

26. Andersson JLR, Graham MS, Zsoldos E, Sotiropoulos SN. Incorporating outlier detection and replacement into a non-parametric framework for movement and distortion correction of diffusion MR images. *Neuroimage*. 2016;141:556-572. doi:10.1016/j.neuroimage.2016.06.058
27. Behrens TEJ, Berg HJ, Jbabdi S, Rushworth MFS, Woolrich MW. Probabilistic diffusion tractography with multiple fibre orientations: What can we gain? *Neuroimage*. 2007;34(1):144-155. doi:10.1016/j.neuroimage.2006.09.018
28. Behrens TEJ, Woolrich MW, Jenkinson M, et al. Characterization and Propagation of Uncertainty in Diffusion-Weighted MR Imaging. *Magn Reson Med*. 2003;50(5):1077-1088. doi:10.1002/mrm.10609
